# Staurosporine as a Potential Treatment for *Acanthamoeba* Keratitis Using Mouse Cornea as an Ex Vivo Model

**DOI:** 10.3390/md22090423

**Published:** 2024-09-18

**Authors:** Rubén L. Rodríguez-Expósito, Ines Sifaoui, Lizbeth Salazar-Villatoro, Carlos J. Bethencourt-Estrella, José J. Fernández, Ana R. Díaz-Marrero, Robert Sutak, Maritza Omaña-Molina, José E. Piñero, Jacob Lorenzo-Morales

**Affiliations:** 1 Instituto Universitario de Enfermedades Tropicales y Salud Pública de Canarias (IUETSPC), Universidad de La Laguna (ULL), Avda. Astrofísico Fco. Sánchez, S/N, 38203 San Cristóbal de La Laguna, Tenerife, Spain; rrodrige@ull.edu.es (R.L.R.-E.); isifaoui@ull.edu.es (I.S.); cbethene@ull.edu.es (C.J.B.-E.); jpinero@ull.edu.es (J.E.P.); 2 Departamento de Obstetricia y Ginecología, Pediatría, Medicina Preventiva y Salud Pública, Toxicología, Medicina Legal y Forense y Parasitología, Universidad de La Laguna, 38203 San Cristóbal de La Laguna, Tenerife, Spain; 3 Centro de Investigación Biomédica en Red de Enfermedades Infecciosas (CIBERINFEC), Instituto de Salud Carlos III, 28220 Madrid, Spain; 4 Departamento de Infectómica y Patogénesis Molecular, Centro de Investigación y de Estudios Avanzados del Instituto Politécnico Nacional, Ciudad de Mexico 07360, Mexico; bioagam@hotmail.com; 5 Instituto Universitario de Bio-Orgánica Antonio González (IUBO AG), Universidad de La Laguna (ULL), 38203 San Cristóbal de La Laguna, Tenerife, Spain; jjfercas@ull.edu.es (J.J.F.); adiazmar@ull.edu.es (A.R.D.-M.); 6 Departamento de Química Orgánica, Universidad de La Laguna (ULL), 38203 San Cristóbal de La Laguna, Tenerife, Spain; 7 Instituto de Productos Naturales y Agrobiología (IPNA), Consejo Superior de Investigaciones Científicas (CSIC), 38203 San Cristóbal de La Laguna, Tenerife, Spain; 8 Department of Parasitology, Faculty of Science, Charles University, BIOCEV, 252 50 Vestec, Prague, Czech Republic; robert.sutak@natur.cuni.cz; 9 Facultad de Estudios Superiores Iztacala, Medicina, National Autonomous University of Mexico (UNAM), Tlalnepantla 54090, Mexico

**Keywords:** *Acanthamoeba*, ex vivo, mouse cornea, staurosporine, proteomic analysis, PCD

## Abstract

*Acanthamoeba* is a ubiquitous genus of amoebae that can trigger a severe and progressive ocular disease known as *Acanthamoeba* Keratitis (AK). Furthermore, current treatment protocols are based on the combination of different compounds that are not fully effective. Therefore, an urgent need to find new compounds to treat *Acanthamoeba* infections is clear. In the present study, we evaluated staurosporine as a potential treatment for *Acanthamoeba* keratitis using mouse cornea as an ex vivo model, and a comparative proteomic analysis was conducted to elucidate a mechanism of action. The obtained results indicate that staurosporine altered the conformation of actin and tubulin in treated trophozoites of *A. castellanii.* In addition, proteomic analysis of treated trophozoites revealed that this molecule induced overexpression and a downregulation of proteins related to key functions for *Acanthamoeba* infection pathways. Additionally, the ex vivo assay used validated this model for the study of the pathogenesis and therapies of AK. Finally, staurosporine eliminated the entire amoebic population and prevented the adhesion and infection of amoebae to the epithelium of treated mouse corneas.

## 1. Introduction

Free-living amoebae are ubiquitous single-celled living organisms isolated from multiple habitats including water and soil. Until present, six different genera have been reported to be pathogenic to humans and animals, which the most common being *Acanthamoeba* genus. This amoeba could cause a threatening eye infection known as *Acanthamoeba* keratitis (AK): a rare eye infection commonly associated with contact lens wearers [1,2]. Although *Acanthamoeba* keratitis constitutes 2% of the corneal infections, its incidence has been consistently increasing [3]. The worldwide annual prevalence of AK stands at a rate of 2.34 cases per million individuals of all reported clinical cases of microbial keratitis, but it can rise to 1 case in 30,000 in contact lens wearers [4,5]. Notably, published studies confirm the increasing trend in rising AK cases in parallel with the number of contact lens wearers. A national prospective survey conducted through the British Ophthalmic Surveillance Unit in the UK reported a significant increase in the incidence of AK in contact lens wearers from 21.14 in 1997/1998 and 17.53 in 1998/1999 to 26.94 in 2015 [6].

Actual treatment protocols are based on the combination of cationic antiseptics such as polyhexamethylene biguanide (0.02%) or chlorhexidine (0.02%) and aromatic diamidines such as propamidine (0.1%) or hexamidine (0.1%) [7,8,9]. Still, these current therapies are not fully effective in eliminating the parasite because of their variable efficacy among different genotypes, the appearance of highly resistant cyst form, or due to their toxicity generated by a prolonged administration. In addition, a delay in correct diagnosis in most clinical cases implies an increased risk of disease progression and the infection of deeper layers of the corneal epithelium. Therefore, early AK detection is critical; however, AK cases are handled in clinics for other causes, such as bacterial or viral infections due to misdiagnosis, resulting in the worsening of AK infections [9,10]. This leads to an urgent need to discover new drugs and/or drug posology.

Compounds with marine origin have proven to be an interesting source for the development of novel antiparasitic therapies. In this sense, indolocarbazole alkaloids have received the attention of researchers due to their natural abundance, wide structural chemical variety, and broad spectrum of biological activities [11]. The staurosporine alkaloid was first identified from the bacteria *Streptomyces staurosporeus* in 1977 [12]. This molecule and its analogs are known as potent protein kinase C inhibitors. Beside this, these indolocarbazoles displayed notably antitumor, antimicrobial, antifungal, antiviral, and antiparasitic activities [11,13,14,15,16]. In previous studies, we demonstrated that staurosporine inhibits *Acanthamoeba* growth and induces programmed cell death (PCD) in trophozoites of *A. castellanii* Neff [17,18].

On the one hand, in vitro assays are essential for drug discovery against the present infection, because they are simple, reproductible and much more economical than the in vivo assay, but still inefficient for predicting the drug’s action inside the host organism. On the other hand, in vivo assays are more clinically relevant, and the drug effect and side effect could be much more accurate, yet in vivo assays are much more expensive and harder to control as they include a multitude of variables. Ex vivo assays are important preclinical tools between in vitro and in vivo assays. In these ex vivo systems, the cytoarchitecture and intracellular connections and metabolic processes could be conserved, mimicking the in vivo environment [19]. Rabbits, hamsters, and mice were used as models for the in vivo assay of *Acanthamoeba* Keratitis [20,21]. To the best of our knowledge, only hamster, porcine, and mouse corneas have been used as animal ex vivo models for *Acanthamoeba* keratitis [22,23,24,25,26,27]. The aim of the present study was to evaluate staurosporine as potential treatment for *Acanthamoeba* keratitis using mouse cornea as an ex vivo model and to investigate its model of action by comparative proteomic analysis.

## 2. Results

### 2.1. Staurosporine Induced Structural Damage of the Acanthamoeba Cytoskeleton

**Figure 1:**
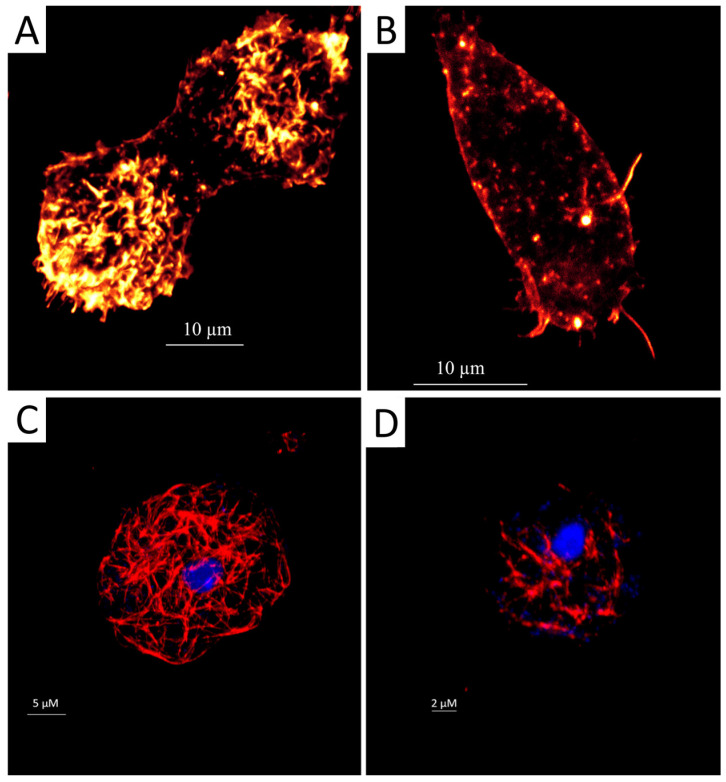
Evaluation of the effect of IC_90_ of staurosporine on the actin and tubulin cytoskeleton of *Acanthamoeba castellanii* trophozoites for 24 h. The phalloidin-TRITC dye stains the polymerised actin cytoskeleton showing the normal organization of the networks with an orange fluorescence in the negative control cells (**A**); scale bar represents 10 µm. Treated cells emitted a lower orange fluorescence, and trophozoites showed disorganized and degraded acanthopodium (**B**); scale bar represents 10 µm. Tubulin antibodies bind to microtubules and stain them in control cells showing an intense red fluorescence and demonstrating a normal conformation (**C**); scale bar represents 5 µm. However, trophozoites incubated with staurosporine show disorganization or destruction of the tubulin microtubules (**D**); scale bar represents 2 µm. Mounting with DAPI solution for DNA staining shows a blue fluorescence (**C**,**D**). All images (63×) were obtained using an inverted confocal light microscope Leica DMI 4000 B (Deerfield, IL, USA).

In eukaryotic cells, the cytoskeleton is mainly composed of proteins like microfilaments, microtubules, and intermediate fibers. Microfilaments, composed essentially of actin filaments, play a crucial role in cellular motility and cell interaction with the extracellular and intracellular environment [28]. In *Acanthamoeba*, targeting the actin network could prevent infection by inhibiting adhesion and cyst formation. A staining using the conjugate phalloidin-TRITC revealed the extent of damage induced by staurosporine on the distribution of actin cytoskeleton: treated cells emitted lower fluorescence, and we observed partly degraded and disorganized acanthopodium, reflecting on the lower expression of actin protein (Figure 1A,B).

As for the microtubules that are mainly composed of tubulin, they have been involved in motility, intracellular transport, and cell division [29]. Inhibiting this protein could prevent cell growth and has been suggested as a good target for amoebicidal agents. An indirect immunofluorescence assay for tubulin detection was conducted. After 24 h of incubation with the staurosporine, we observed a decrease in cell shape with a uniformly distributed tubulin network. The affected cells emit lower fluorescence than the untreated cells (Figure 1C,D).

### 2.2. Proteomic Analysis

**Figure 2:**
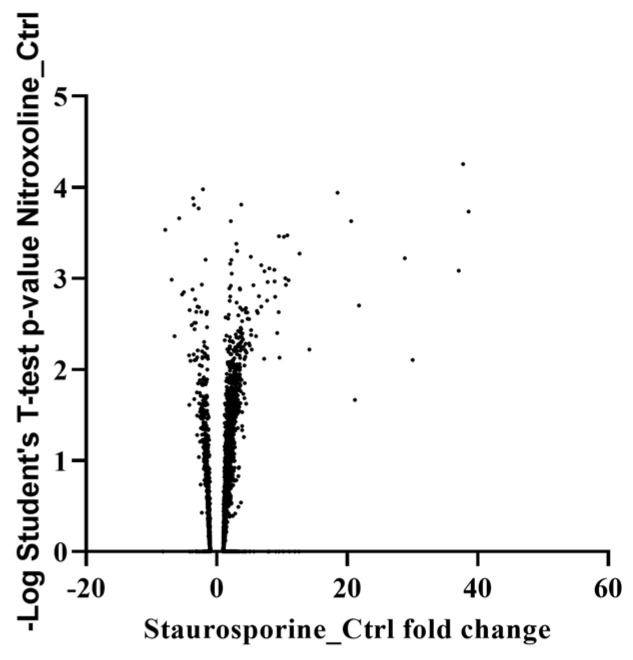
Volcano graph expressing a logarithmic Student’s *t*-test *p*-value as a function of staurosporine protein control fold change.

To comprehend the effect of staurosporine on *Acanthamoeba*, a mass spectrometry-based proteomic approach was conducted. Two groups of cells at the trophozoite stage of *Acanthamoeba castellanii* were prepared: untreated and treated cells with IC_50_ of the present molecule. After 24 h of treatment, total proteins were extracted. The proteomic profiling of both cultures resulted in the identification of 4566 proteins (Supplementary Materials, Protein analysis). Compared to untreated cells, the proteomic analysis revealed that a total of 812 proteins were differentially expressed at least twofold, in which 97 and 715 proteins were downregulated and upregulated, respectively (Figure 2). The selected affected proteins discussed below are listed in Table 1.

### 2.3. Ex Vivo Assay in Mouse Corneas Infected with A. castellanii and Treated with Staurosporine

**Figure 3:**
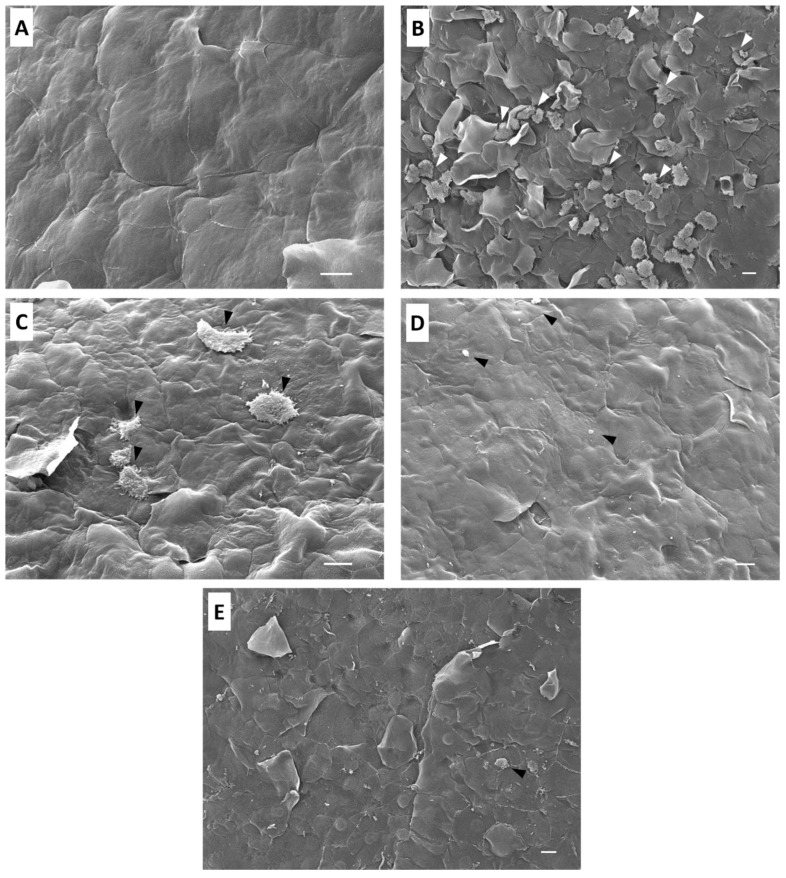
Scanning electron microscopy analysis of the effect of *Acanthamoeba castellanii* trophozoites and/or staurosporine on mouse corneas during 3 h. The co-incubation of mouse corneas with staurosporine at IC_90_ shows no evidence of damage or cell disorganization (**A**); Group 1, scale bar represents 10 µm. Mouse corneas co-cultured with trophozoites of *Acanthamoeba castellanii* (**B**); scale bar represents 10 µm; Group 2 presented de-epithelization of the corneal epithelium by the amoebic infection penetrating between the junctions of the epithelial cells (white arrows). Corneas co-incubated with trophozoites of *Acanthamoeba castellanii* pretreated with staurosporine 30 min prior (**C**); scale bar represents 10 µm; Group 3 showed that fewer trophozoites adhered to the corneal epithelium (black arrows), which did not migrate toward the inner layers of the corneal epithelium; therefore, the damage was very limited. In the corneas with simultaneous infection and treatment with staurosporine (**D**)—Group 4, scale bar represents 10 µm—the cellular debris of *Acanthamoeba castellanii* trophozoites was observed (black arrows), and the corneal epithelium showed no cell damage or signs of amoebic invasion. Corneas co-cultured with trophozoites and treated with staurosporine 30 min after infection (**E**); scale bar represents 10 µm; Group 5 showed that no-healthy cells adhered to the corneal surface (black arrow), and the corneal epithelium showed signs of early stages of amoebic infection. Images were obtained using a JEOL-JSM 7100F scanning electron microscope (JEOL Ltd., Tokyo, Japan).

Scanning electron microscopy was used to analyze the effect of staurosporine in the mouse corneas infected with trophozoites of *Acanthamoeba castellanii.* Mouse corneas incubated alone with staurosporine at IC_90_ for *A. castellanii* presented no corneal injury. Furthermore, epithelial cells showed a normal conformation and structure (Figure 3A). In the group of mouse corneas cultured alone with trophozoites, amoebas adhered to the corneal surface and penetrated the junctions of the epithelial cells of the cornea, taking advantage of these junctions to produce infections of the epithelium (Figure 3B). Contact damage was observed without lysis; corneal epithelium showed de-epithelization and destabilization produced by trophozoites; and then, phagocytosis took place.

The group co-cultured with trophozoites pretreated with the IC_90_ of staurosporine before incubation showed that the epithelium cells were not damaged, and scarce amoebas were observed (Figure 3C). The pre-treatment with staurosporine affected the adhesion of the amoebae to the epithelial cells, and consequently, prevented the amoebae from invading the corneal epithelium. Mouse corneas co-incubated simultaneously with trophozoites and staurosporine at IC_90_ revealed that corneal epithelium was intact without signs of cellular damage. Moreover, no healthy amoebae were observed on the epithelium. The simultaneous treatment with staurosporine eliminated all the amoebas, preventing the amoebic invasion of the corneal epithelium (Figure 3D).

Mouse corneas co-cultured with trophozoites and treated with staurosporine after 30 min of incubation showed no healthy adherent trophozoites on the corneal epithelium. The amoebae presented morphological alterations after staurosporine treatment. The corneal epithelium showed cellular damage caused by the early stages of amoebic, but once staurosporine was administered after 30 min of incubation, it acted directly on the trophozoites, preventing a more severe invasion of the corneal epithelium (Figure 3E).

## 3. Discussion

Staurosporine was first isolated by Omura, S. et al. in 1977 from a Streptomyces strain [12]. Until present, several authors have confirmed its pharmacological activities like hypotensive, antiprotozoal, anticoagulant, and antifungal properties [11,30]. In a previous work, we proved the amoebicidal activity of staurosporine against various *Acanthamoeba* strains [18], showing IC_50_ values of 0.568 ± 0.122 µM and 1.653 ± 0.017 µM in trophozoite and cyst stages of *A. castellanii* Neff, respectively. The approach was based on molecule isolation and bio-guided fractionation from a *Streptomyces sanyensis* extract. In addition, we confirmed that the present indolocarbazole could induce program cell death in *A. castellanii* Neff, evidencing plasma membrane damage, chromatin condensation, the collapse of the ATP level, and mitochondrial membrane potential, as well as increased levels of reactive oxygen species (ROS) [17,18]. In this regard, to confirm the obtained results and to indicate the mechanism of action, we opted to study the effect of the present molecules on the proteomic profile of *Acanthamoeba* and to confirm its amoebicidal activity using an ex vivo approach.

The main objectives of the present work were to first confirm the amoebicidal effect of staurosporine on this clinical strain of *Acanthamoeba castellanii*, following protein expression in the early stage of treatment. Second was to establish a new protocol to study the effects of the amoebicidal drug on an amoebic keratitis murine ex vivo model, which turned out to be efficient since data similar to those observed and described in a previous study in the cornea of hamster (*Mesocricetus auratus*) were obtained.

In our previous work, we highlighted the morphological alterations induced by the staurosporine on *Acanthamoeba* in the early stage of treatment. To corroborate the effect of the present drug on the cytoskeleton of *Acanthamoeba*, a specific staining of actin and tubulin was carried out. After 30 min of incubation with the present drug, we observed a dramatic alteration in the cell cytoskeleton; actin staining revealed the formation of long elongation. Various reports confirm the present findings; Hedberg et al. (1990) reported an alteration in the cytoskeleton of several cell lines, including PTK2 epithelial cells, Swiss 3T3 fibroblasts, and human foreskin fibroblasts, by staurosporine [31], while Xie et al. (2017) observed the formation of filaments in the fungal pathogen *Candida albicans* [32]. In fact, they related the staurosporine-induced filament to a defect in septin ring formation, implicating cell cycle kinases as potential staurosporine targets [32]. As for the microtubules network, we observed that the present drug induces alteration in its rearrangement as a disorganization network with the presence of concentrated points. All those events could be a result of a cell dismantling upon program cell death [33].

The proteomic analysis of cells treated with the staurosporine (IC_50_) for 24 h revealed that various membrane protein kinases were significantly downregulated, including dual specificity protein kinase shkB and Serine/threonine-protein kinase. Those proteins have been described as regulators of the chemotaxis and phagocytosis processes in *Acanthamoeba.* Inhibiting these proteins could reduce the pathogenicity and growth of *Acanthamoeba*. Along with this effect, we noticed the inhibition of cysteine protease implicated in the tissue invasion as well as in the encystation pathway [34].

In treated cells, almost 16% of the total identified proteins were overexpressed. Among those proteins, we observed the upregulation of Profilin. This molecule is implicated in the regulation of actin polymerization and affects the structure of the cytoskeleton. Various reports have confirmed that at lower concentrations, this protein enhances the actin polymerization, while at high concentrations, it would prevent it and trigger the autophagy via the mTOR pathway [35,36]. Among the most upregulated proteins, we found proteins involved in cell survival under oxidative stress.

Although, we have confirmed in vitro the effect of staurosporine on *Acanthamoeba*, these results are still insufficient to scale up to in vivo. For this reason, one of the main objectives of the present study was to establish an ex vivo model to study the effect of drug therapy on AK infection. Omaña-Molina et al. (2004) established an ex vivo model to study the cytopathic effects of *Acanthamoeba castellanii* and *A. polyphaga* on hamster corneas [23]. In this study, the ex vivo assay revealed that in the trophozoites of *Acanthamoeba castellanii* infected the mouse corneas, showing that the amoebas invaded the corneal epithelium, penetrating the junctions of the epithelial cells without lysis process and only phagocyting the detached cells. This type of invasion suggests that *Acanthamoeba* infections are contact-dependent. In this sense, Omaña-Molina et al. (2013) demonstrated that the *A. castellanii* and *A. polyphaga* invasion and disruption of corneal epithelium in the hamster model is performed by the penetration of the amoebae through cell junctions either by the action of proteases and/or a mechanical effect exerted by trophozoites, suggesting that the contact-dependent activity is an important pathogenic mechanism of these strains of *Acanthamoeba* [24]. Wang et al. (2021) studied the crucial role of commensals in mitigating *A. castellanii* pathogenicity [26] using a modified ex vivo mouse model based on the previously reported ex vivo hamster model by Omaña-Molina et al. (2013). This ex vivo mouse study demonstrated that the presence of intact bacteria significantly reduced *A. castellanii* corneal epithelial cell damage. Moreover, in a previous study, Omaña-Molina et al. (2010) co-cultivated *A. castellanii* trophozoites with human corneas and reported that the mechanisms of pathogenicity of amoebic infections were very similar to those in the previous study using a hamster model and our current findings obtained using a mouse model, which validates these animal models for the study of the pathogenesis of AK [23,25,37].

Currently, the agents normally recommended to treat AK need to be administered for a prolonged period, which often results in severe ocular surface toxicity [7,38,39,40,41]. In this study, mouse corneas cultured alone with staurosporine at a concentration of 2.70 ± 0.015 µM revelated that this compound causes no adverse effect on the corneal epithelium compared to other drugs, maintaining the mouse corneal tissues intact after 3 h of incubation. In our previous study, the toxicity effect observed when tested in vitro against a macrophage cell line was low (cytotoxicity concentration value of 50, CC_50_, 8.737 ± 0.718 µM) in comparison to the obtained IC_50_ and IC_90_, with values 4-fold higher than the staurosporine IC_50_ against cysts [18]. Nevertheless, Härtel et al. (2003) reported that staurosporine could induce apoptosis in human corneal epithelial (HCE) cells [42]. HCE cells were incubated in vitro with different concentrations of staurosporine, demonstrating pathological effects after 4 h at 2 µM. In this regard, our ex vivo mouse model has shown that staurosporine with only 30 min pre-treatment affects the adhesion of the amoebae to the corneal epithelium and prevents the trophozoites from invading the corneal tissue. However, a 30 min pre-treatment did not completely eliminate the amoebae population, possibly due to insufficient time for the pre-treatment of the trophozoites. The corneal epithelium was intact in the infected mouse corneas treated simultaneously and 30 min after infection, showing similar results to those of the mouse corneas cultured alone with staurosporine. This compound eliminated the entire amoebic population, preventing the adhesion and infection of amoebae to the epithelium with only 3 h of incubation. Therefore, this study demonstrated that staurosporine could be used to develop a new line of eye drops for the treatment of superficial AK or early stages of amoebic infection.

Nevertheless, our study had limitations. For example, for patients with AK in the chronic stage of the disease, where the infection is located at deeper levels of the corneal epithelium, further studies are needed to determine whether the effect of staurosporine could be observed deeper in the corneal tissue and completely eliminate the amoebic infection, as well as to determine the possibility of toxic effects in longer treatments.

## 4. Materials and Methods

### 4.1. Acanthamoeba Strain

Trophozoites of *A. castellanii* (genotype T4) were isolated from a clinical case of AK at “Asociación para evitar la ceguera en México”, Luis Sánchez Bulnes Hospital, Mexico City. The strain was reported to be invasive in the GAE murine model [43,44,45]. The strain was grown axenically in PYG medium (0.75% (*w*/*v*) proteose peptone, 0.75% (*w*/*v*) yeast extract, and 1.5% (*w*/*v*) glucose) containing 40 μg gentamicin mL^−1^ (Biochrom AG, Cultek, Granollers, Barcelona, Spain) at 26 °C. After 72 h of incubation (the end of the growth logarithmic phase), the culture was centrifuged (2500 rpm during 5 min), and trophozoites were harvested for the subsequent assays.

### 4.2. In Vitro Assay

#### 4.2.1. Fluorescent Staining of Actin Distribution

For direct fluorescent staining, trophozoites of *A. castellanii* were treated first with the IC_90_ (2.70 ± 0.015 µM) of the staurosporine with a cell concentration of 2.5 × 10^5^ mL^−1^. After 30 min of incubation, cells were fixed with formaldehyde and deposited on a pre-coated coverslip. Later, cells were treated with Triton (0.1%) for 30 min followed by Phalloidin–tetramethylrhodamine B isothiocyanate (Phalloidin-TRITC; Sigma-Aldrich, Madrid, Spain) for another 30 min at room temperature. Finally, cells were washed with PBS and later examined by *Z*-stack imaging using an inverted light confocal microscope Leica DMI 4000 B with LAS X software (Version 5.2.2), a 532 nm laser, and a Leica HCX PL Apo 63× Oil Objective (Leica Microsystems, Wetzlar, Germany). Untreated cells were considered the negative control.

#### 4.2.2. Immunofluorescence Staining of the Tubulin of Acanthamoeba Trophozoites

The immunofluorescence staining of tubulin was conducted using the immunofluorescence staining procedure of the manufacturer (Sigma-Aldrich) with slight modifications. Briefly, trophozoites (2.5 × 10^5^ cells mL^−1^) were first treated with the IC_90_ of staurosporine for 30 min. In total, 50 µL of the cell suspension was placed on a gelatine precoated coverslip for 30 min. Later, it were fixed with paraformaldehyde (4%). After 15 min, the cells were treated with Triton (0.3%) for 10 min followed by 3 washes with PBS 1×. At this stage, the cells were treated with 5% BSA in PBS 1×/150 mM sucrose for 30 min and washed with glycine 100 mM in PBS 1× for 5 min. Later, the trophozoites were incubated with the first anti-tubulin antibody 1:2000 for 2 h at room temperature (Monoclonal Anti-α-Tubulin antibody produced in mouse, Sigma-Aldrich, Madrid, Spain). After 3 washes with PBS 1×, the cells were incubated with the second antibody labeled with Alexa 594 (1:500) for 1 h at room temperature in darkness (Goat anti-Mouse IgG (H+L) Highly Cross—Adsorbed Secondary Antibody, Alexa Fluor Plus 594; Thermo Fisher Scientific, Rockford, IL, USA). Finally, the cells were washed with PBS 1× and mounted in DAPI (4′,6-Diamidino-2-phenylindole dihydrochloride; Sigma-Aldrich; Madrid, Spain) containing mounting solution. Three-dimensional and maximum projection imaging of the trophozoites were performed by Z-stack imaging using an inverted light confocal microscope Leica DMI 4000 B, LAS X software, a 405 nm laser, a 532 nm laser, and a Leica HCX PL Apo 63× Oil Objective.

### 4.3. Proteomic Analysis

Comparative label-free proteomic analysis was conducted as described in our recent study [46]. In total, 10^6^ cells of *A. castellanii* were treated with the IC_50_ (0.568 ± 0.122 µM) of staurosporine for 24 h and washed 1 time with PBS, and the pellets were subjected to further processing. Untreated cells were prepared as a control group. Both groups were prepared in three biological replicates. Protein identification was made using the data base of https://www.uniprot.org/ (accessed on 1 October 2023).

### 4.4. Ex Vivo Infection

#### Co-Incubation and Interaction of *A. castellanii* Trophozoites with BALB/c Mice Cornea

A total of 12 pathogen-free male BALB/c (*Mus musculus*) mice were used, with an average age of 21 to 28 days and an average body weight of 35 g. The experiments were based on the protocol previously described for hamster cornea (*Mesocricetus auratus*) [23,24,25], were approved by the Research Ethics and Animal Welfare Committee of the University of La Laguna, included in the project known as the Evaluation of the in vivo amoebicidal activity of eye drops containing active ingredients administered via the ocular route, with reference number CEIBA2021-3074.

The corneas of each mouse were removed and processed. Then, the corneas were placed in 96-well polystyrene plates and were washed with 1× PBS twice. Five experimental groups were established in the 96-well plates. Group 1 included corneas incubated alone with staurosporine at IC_90_ of *A. castellanii*. Corneas in Group 2 were co-cultured alone with 10^6^ *Acanthamoeba castellanii* trophozoites. Corneas in Group 3 were co-incubated with pretreated *A. castellanii* with the IC_90_ of staurosporine for 30 min. Group 4 consisted of corneas co-incubated simultaneously with trophozoites and with IC_90_ of staurosporine. Corneas of Group 5 were co-cultured alone with *A. castellanii* trophozoites, and after 30 min of incubation, amoebas were treated with the IC_90_ of staurosporine. The plates with all groups of mouse corneas were incubated in a humidity chamber at 36 °C during 3 h.

### 4.5. Scanning Electron Microscopy

After co-incubation, all groups of corneas were fixed at room temperature with 2.5% glutaraldehyde in 0.1 M sodium cacodylate buffer, dehydrated in increasing concentrations of ethanol, and critical-point-dried with liquid CO_2_ using a Samdri 780 apparatus (Tousimis, Rockville, MD, USA). Then, the corneas were coated with a thin layer (30 nm) of gold in a JEOL-JFC I 100 ion-sputtering device. Finally, all groups of corneas were observed using a JEOL-JSM 7100F scanning electron microscope (JEOL Ltd., Tokyo, Japan) [24].

## 5. Conclusions

In summary, staurosporine induced structural alterations in the actin and tubulin cytoskeleton in trophozoites of *Acanthamoeba castellanii*. In addition, the proteomic analysis of treated trophozoites with staurosporine showed that various membrane protein kinases and cysteine proteases were significantly downregulated, mainly involved in the pathogenicity, growth, tissue invasion, and encystation pathway of *Acanthamoeba.* Furthermore, almost 16% of the total identified proteins were overexpressed. We observed the upregulation of Profilin, which is involved in the regulation of actin polymerization, affecting the structure of the cytoskeleton. On the other hand, the mechanisms of the pathogenicity of amoebic infection reported in previous ex vivo investigations were very similar to our findings using a mouse model, which validate this animal model for the study of the pathogenesis and therapies of AK. Besides this, staurosporine eliminated the entire amoebic population and prevented amoeba adhesion and infection to the epithelium of mouse corneas, demonstrating that staurosporine could be used to develop a new line of eye drops for the treatment of superficial AK or early stages of amoebic infections.

## Figures and Tables

**Table 1:** Changes in the levels of selected proteins upon staurosporine treatment at IC_50_ for 24 h.

Gene ID	Product	Fold Change Staurosporine/Control
ACA1_376810	Universal stress domain containing protein	N.d. *. in control
ACA1_146430	Catalase	8.87
ACA1_392960	Universal stress domain containing protein	3.44
ACA1_387720	Universal stress domain containing protein	2.92
ACA1_076370	Profilin	2.69
ACA1_265580	Manganese and iron superoxide dismutase	2.67
ACA1_031660	Catalase	2.59
ACA1_062010	Universal stress domain containing protein	2.41
ACA1_236670	Universal stress protein (USP) family protein	2.38
ACA1_045080	Thioredoxin, putative	2.28
ACA1_054650	Glutathione transferase family protein	2.03
ACA1_095580	Serine/threonine kinase	−2.16
ACA1_288400	Dual specificity protein kinase shkB, putative	−3.73
ACA1_159080	Papain family cysteine protease subfamily protein	N.d. in Staurosporine

* N.d.: not detected.

## Data Availability

The original contributions presented in the study are included in the article/Supplementary Materials; further inquiries can be directed to the corresponding authors.

## Supplementary Materials

The following supporting information can be downloaded at https://www.mdpi.com/article/10.3390/md22090423/s1, File S1: Experimental conditions for the proteomic analysis of *Acanthamoeba* trophozoites treated with staurosporine.
